# Microstructure Evolution and Constitutive Model of Spray-Formed 7055 Forging Aluminum Alloy

**DOI:** 10.3390/ma18174108

**Published:** 2025-09-01

**Authors:** Yu Deng, Huyou Zhao, Xiaolong Wang, Mingliang Cui, Xuanjie Zhao, Jiansheng Zhang, Jie Zhou

**Affiliations:** 1 College of Materials Science and Engineering, Chongqing University, Chongqing 400044, China; dyu0505@163.com (Y.D.);; 2 China National Erzhong Group Deyang Wanhang Die Forging Co., Ltd., Deyang 618000, China; 3 Chongqing Jiepin Technology Co., Ltd., Chongqing 400050, China

**Keywords:** spray-formed 7055 aluminum alloy, constitutive model, hot deformation behavior, microstructure

## Abstract

The thermal deformation behaviour of a spray-formed 7055 as-forged aluminium alloy was studied using isothermal hot-press tests under different deformation conditions (strain rates of 0.01, 0.1, 1, and 10 s^−1^, temperatures of 340, 370, 400, 430, and 460 °C). An Arrhenius constitutive model was developed using flow stress data corrected for friction and temperature, yielding a correlation coefficient (R) of 0.9877, an average absolute relative error (AARE) of 4.491%, and a deformation activation energy (Q) of 117.853 kJ/mol. Processing maps integrating instability criteria and power dissipation efficiency identified appropriate processing parameters at 400–460 °C/0.08–0.37 s^−1^. Furthermore, this study investigated how strain rate and temperature influence microstructural evolution. Microstructural characterization revealed that both dynamic recovery (DRV) and dynamic recrystallization (DRX) occur simultaneously during thermal deformation. At low temperatures (≤400 °C), DRV and continuous dynamic recrystallization (CDRX) dominated; at 430 °C, deformation microstructures and recrystallized grains coexisted, whereas abnormal grain growth prevailed at 460 °C. The prevailing mechanism of dynamic softening was influenced by the applied strain rate. At lower strain rates (≤0.1 s^−1^), discontinuous dynamic recrystallization (DDRX) was the primary mechanism, whereas CDRX became dominant at higher strain rates (≥1 s^−1^), and dislocation density gradients developed within adiabatic shear bands at 10 s^−1^.

## 1. Introduction

Aluminum alloys are widely applied in aviation, aerospace, and diverse transportation equipment manufacturing due to exceptional strength-to-weight ratio, favorable corrosion resistance, outstanding formability and machinability [1]. However, conventional aluminum alloys often suffer from high-temperature strength degradation, limited fatigue resistance, and susceptibility to stress corrosion cracking in corrosive environments, etc., 7055 aluminum alloy represents the high-performance aluminum alloy series, which is significantly superior to conventional high-strength aluminum alloys such as 7075 and 2024 in terms of strength, toughness, and resistance to stress corrosion, is a key material for the load-bearing components of the main body of an aircraft [2,3]. However, the 7055 alloy produced by conventional ingot metallurgy still faces some inherent constraints brought about by its high Zn and Cu composition, including insufficient plasticity at room temperature and hot working conditions, low fracture toughness, narrow hot working interval, and severe macro-segregation and formation of coarse grain boundary phases, which degrade the performance of the components [2]. So as to solve the above process and material bottlenecks, the spray forming technique has been adopted for producing 7055 aluminum alloy. Compared with traditional 7055 aluminum alloy, the spray-formed variant exhibits more equiaxed and refined grains, reduced segregation, enhanced impact properties, and superior specific strength [4,5]. However, spray-formed alloys typically contain porosity, necessitating homogenization treatment followed by further densification through processes such as extrusion and rolling [6,7,8,9,10]. Prior to employing spray-formed alloys for large-scale aerospace die forging, introducing pre-deformation during billet preparation can further improve material densification and microstructural homogeneity, thereby effectively enhancing the quality of forged components. During die forging, the microstructure and properties of forgings are influenced by deformation parameters. Inappropriate deformation conditions may severely compromise forging quality, inducing defects such as cracks and folds [11,12]. Therefore, constructing a constitutive model capable of characterizing the relationship between rheological stress and process parameters, and systematically investigating the effects of process parameters on the evolution of material microstructure, holds significant research value.

Isothermal hot-press tests are typically employed to investigate optimal processing conditions in actual manufacturing processes and to provide reference parameters for them. Shao et al. [13] studied the flow behavior of spray-formed 7055 aluminum alloy ingots and pointed out that the dynamic recrystallization (DRX) mechanism is dependent on the thermal deformation conditions. Lin et al. [14] explored the hot deformation behavior of spray-formed 7055 aluminum alloy extruded plates in the different deformation conditions. They also employed the GA-BP-ANN method to establish a high-precision prediction model and a three-dimensional machining map. Zhang et al. [15] studied the hot deformation characteristics of homogenized spray-formed Al-Zn-Mg-Cu alloys. They systematically analyzed the effects of different deformation conditions on the softening mechanism, demonstrating that continuous dynamic recrystallization (CDRX) occurs at low temperatures or high strain rates.

According to the literature, existing research on spray-formed aluminum alloys with ultra-high strength has primarily focused on material behavior during homogenization, extrusion, and solution treatment. However, studies on the thermal deformation behavior and microstructural evolution exhibited by these alloys during hot working—specifically after preforming deformation and prior to full forging—remain insufficient. This knowledge gap makes it challenging to simultaneously ensure both the forming quality and final performance metrics of spray-formed aluminum alloys with ultra-high strength meet application requirements during actual forging production.

This study employs isothermal hot-press testing to systematically investigate the hot deformation behavior and DRX mechanism of spray-formed 7055 as-forged aluminum alloy. Using corrected flow stress data, an Arrhenius constitutive model was developed. By integrating work consumption and instability maps, a hot working map was established. Furthermore, the implication of temperature and strain rate on microstructural evolution during hot deformation is examined.

## 2. Materials and Experimental Method

### 2.1. Experimental Materials and Methods

The research material was spray-formed 7055 as-forged aluminum alloy plate supplied by Deyang Wanhang Die Forging Co., Ltd., in Deyang, China, subjected to free forging. Table 1 shows its chemical composition. The spray-formed billet was extruded, subsequently free forged at 430 °C, and then cools to room temperature.

**Figure 1:**
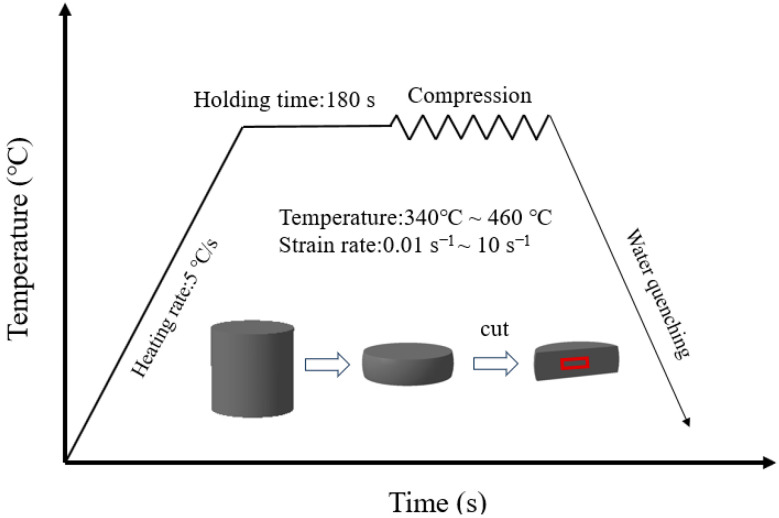
Experimental flowchart and sample observation area during the hot-press process.

Cylindrical specimens with a height of 12 mm and a diameter of 8 mm were obtained from the plate using the wire-cutting method for experimental purposes. Hot-press tests using the Gleeble-3500 thermomechanical simulation apparatus (Data Sciences International, Inc., St. Paul, MN, USA); the resulting flow curves are presented in Figure 1. Each specimen was resistance-heated at 5 °C/s to one of five predefined temperatures: 340 °C, 370 °C, 400 °C, 430 °C, or 460 °C. Following heating, a 3 min isothermal hold was implemented to achieve a uniform temperature distribution. The samples were then compressed at constant strain rates of 0.01 s^−1^, 0.1 s^−1^, 1 s^−1^, or 10 s^−1^. All deformations were interrupted upon reaching a true strain of 0.91. To retain the microstructure, the compressed specimens were immediately water-quenched at room temperature. To reduce the impact of friction during compression, lathe marks on the specimen surface were removed prior to testing, and graphite sheets were added at both ends as lubricants.

### 2.2. Microstructure Observation

This work employed electron backscatter diffraction (EBSD; TESCAN MIRA 3 SEM, TESCAN, Brno, Czech Republic) to analyze the microstructure. Due to the inherent heterogeneity in strain distribution within the material during thermal deformation, distinct microstructural morphologies emerge across different regions of the compressed specimen. To ensure consistency in analytical results, all microscopic observations were uniformly conducted in the central region of the specimen. In this work, recrystallized grains and recovered regions were differentiated using color mapping based on intragranular misorientation. Based on the EBSD misorientation data, grain boundaries were categorized as either high-angle (HAGBs, >15°) or low-angle (LAGBs, 2°–15°).

**Figure 2:**
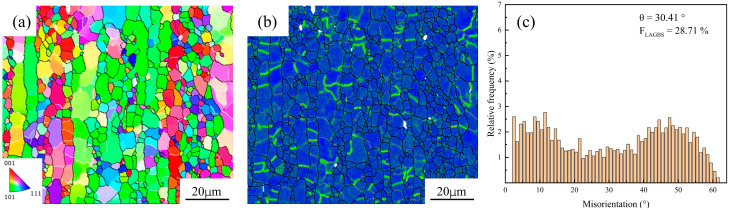
Initial microstructure of spray-formed 7055 as-forged aluminum alloy. (**a**) IPF maps; (**b**) KAM maps; (**c**) MAD maps.

Figure 2 shows the EBSD analysis of the sample before hot compression, including the Inverse Pole Figure (IPF) map, Kernel Average Misorientation (KAM) distribution, and the Misorientation Angle Distribution (MAD) histogram. It can be observed that the fraction of the LAGBs was 28.71%, and the average misorientation angle was 30.41°. The microstructure was mainly characterized by elongated grains, with numerous fine equiaxed grains distributed along their boundaries, suggesting that DRX had already taken place at this stage.

## 3. Results and Discussion

### 3.1. Analysis of Stress–Strain Curves During Thermal Deformation Processes

During deformation, although graphite sheets were used to reduce friction, friction remains unavoidable. This leads to inhomogeneous deformation in the specimen. Furthermore, the barreling effect generated during compression further exacerbates friction between the dies and the specimen. Therefore, friction correction [16] must be applied to the obtained stress–strain curves. This study adopted the friction correction method proposed by Ebrahimi et al. [17].

Furthermore, the deformation heating effect causes the actual deformation temperature of the specimen to fluctuate within a certain range, deviating from the preset temperature. This deviation means that the measured yield stresses that are lower than actual values Therefore, a temperature correction must be applied to the stress–strain curves. The correction formula is as follows [18]:

(1) $$\sigma_{c}=\sigma_{f}+∆\sigma$$

(2) $$∆\sigma =∆T\frac{d\sigma }{dT}{|}_{\varepsilon ,\dot{\varepsilon }}$$

(3) $$∆T=\frac{\left(0.95𝜂\int \sigma d\varepsilon \right)}{\left(\rho C_{p}\right)}$$

(4) $$𝜂=\left\{\begin{array}{c} 0\;\;\;\;\;\;\;\;\;\;\;\;\;\;\;\;\;\;\;\;\;\;\;\;\;\;\;\;\;\;\;\;\;\;\;\;\;\;\;\;\;\;\;\;\;\;\;\;\;\;\;\;\;\;\;\;\;\dot{\varepsilon }\leq 0.001s^{-1}\;\;\\ 0.316{log}_{10}\dot{\varepsilon }+0.95\;\;\;\;\;\;\;\;\;\;\;\;\;\;\;\;0.001s^{-1}\leq \dot{\varepsilon }\leq 1s^{-1}\\ 0.95\;\;\;\;\;\;\;\;\;\;\;\;\;\;\;\;\;\;\;\;\;\;\;\;\;\;\;\;\;\;\;\;\;\;\;\;\;\;\;\;\;\;\;\;\;\;\;\;\;\;\;\;\;\;\;\;\;\;\;\;\dot{\varepsilon }\geq 1s^{-1}\; \end{array}\right.$$

Here, ΔT represents the adiabatic temperature rise (unit: °C), β denotes the correction factor, ρ indicates the material density (unit: g/cm^3^), and Cp is the specific heat capacity of the material (unit: J g^−1^ K^−1^). The coefficient 0.95 reflects the proportion of mechanical work converted into heat, while the remaining energy primarily influences microstructural evolution.

**Figure 3:**
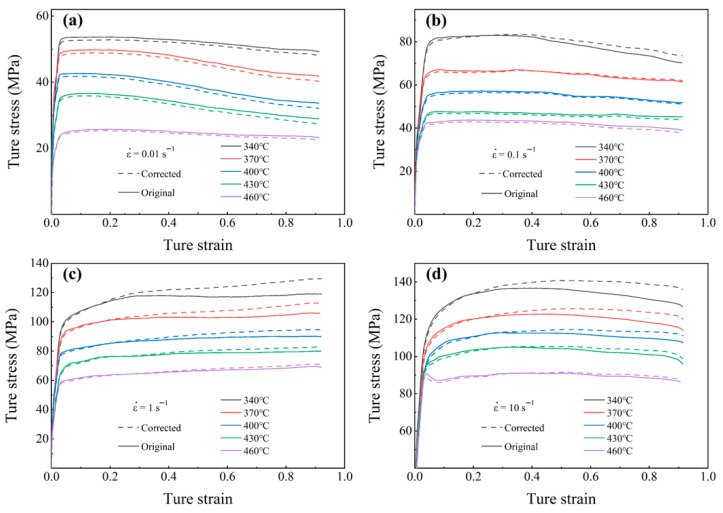
True stress–strain curves before and after friction and temperature correction, (**a**) 0.01 s^−1^; (**b**) 0.10 s^−1^; (**c**) 1.00 s^−1^; (**d**) 10.00 s^−1^.

Figure 3 shows the flow stress curves of spray-formed 7055 forged aluminum alloy before and after friction and temperature correction. Similar trends are evident in both the uncorrected and corrected stresses. Reductions in temperature and strain rate leads to the decreasing trend for peak stress. Deviation between uncorrected and corrected values due to the combined effects of temperature and strain rate.

At low strain rates, the corrected stress is lower than the uncorrected value, suggesting that the strengthening effect caused by friction dominates over the thermal softening effect resulting from temperature rise. With increasing strain rate, however, pronounced deformation heating enhances material softening, which effectively counteracts the work-hardening attributed to friction [19]. Therefore, the corrected stress becomes higher than the uncorrected value under high strain rate conditions. At a fixed strain rate, the difference in stress before and after calibration gradually decreases as the deformation temperature increases. This is due to friction having a reduced influence at higher temperatures [20]. Furthermore, the difference between the flow stresses before and after calibration increases with increasing strain, primarily attributable to the barrel effect that emerges during the later stages of deformation. This effect exacerbates friction at the die–specimen interface, thereby magnifying its contribution to the flow stress.

Further analysis of each modified flow stress curve reveals that flow stress exhibits a rapid initial increase during the early stages of deformation. This is because the rapid accumulation of dislocations and their subsequent entanglement, which hinders further plastic deformation and leads to work hardening. After reaching the peak stress, the flow stress behavior demonstrates a strong dependence on temperature and strain rate. Observe Figure 3c,d, under conditions of low temperatures and high strain rates, the flow stress curve remains steady or gradually increases after the peak stress. This indicates that work hardening dominates the deformation process at this stage, while softening mechanisms are either insufficiently activated or unable to keep pace [21]. Conversely, according to Figure 3a,b at elevated temperatures and reduced strain rates, the curve gradually decreases and stabilizes after the peak. This suggests that dynamic softening mechanisms are fully activated [22]. Notably, a sudden stress drop occurred in the specimen under conditions of 430 °C/10 s^−1^ (see Figure 3d). This is due to the occurrence of DRX, accompanied by dislocation annihilation [13].

### 3.2. Constitutive Equation Establishment

This study employed adjusted flow stress data to establish the Arrhenius constitutive model proposed by Cyrus and Taggart, which describes the interrelationship among flow stress, deformation temperature, and strain rate, as expressed in the following equations [23,24]:

(5) $$\dot{\dot{\varepsilon }=AF\left(\sigma \right)}exp\left(-\frac{Q}{RT}\right)$$

(6) $$\left(\sigma \right)=\left\{\begin{array}{c} \sigma^{B}\;\;\;\;\;\;\;\;\;\;\;\;\;\;\;\;\;\;\;\;\;\;\;\;\;\;\;\;\;\;\;\;\;\;\;\;\;\;\left(\alpha \sigma <0.8\right)\\ exp\left(\beta \sigma \right)\;\;\;\;\;\;\;\;\;\;\;\;\;\;\;\;\;\;\;\;\;\;\;\;\;\;\;\;\;\left(\alpha \sigma >1.2\right)\\ {\left[sinh\left(\alpha \sigma \right)\right]}^{n}\;\;\;\;\;\;\;\;\;\;\;\;\;\;\;\;\;\;\;\;\;\;\;\;\left(for\;all\;\sigma \right) \end{array}\;\;\;\right.$$

In this formulation, $\dot{\varepsilon }$ is the strain rate (s^−1^), σ denotes the flow stress (MPa), Q stands for the activation energy (kJ/mol), R refers to the universal gas constant, and T indicates the absolute temperature (K). The symbols β, B, n, and A denote material constants, with α defined as β/B_1_.

Logarithmic transformations were performed on both sides of the equation under multiple stress conditions to solve for the A, α, B, n, and β. The resulting logarithmic expressions are given below:

(7) $$ln\left(\dot{\varepsilon }\right)=\left\{\begin{array}{c} lnA+Bln\sigma -Q/RT\\ lnA+\beta \sigma -Q/RT \end{array}\;\;\;\to \;\right.\;\begin{array}{c} B=\frac{\partial ln\dot{\varepsilon }}{\partial ln\sigma }{|}_{T}\\ \beta =\frac{\partial ln\dot{\varepsilon }}{\partial \sigma }{|}_{T} \end{array}$$

**Figure 4:**
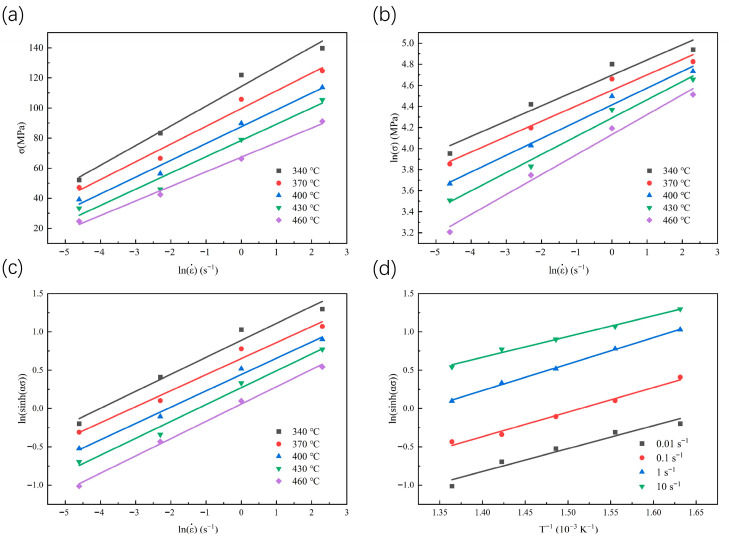
Flow stress at a strain of 0.4 as a function to strain rate and deformation temperature. (**a**) $\sigma -\ln\dot{\varepsilon }$; (**b**) $\ln\sigma -\ln\dot{\varepsilon }$; (**c**) $\ln[sinh\left(\alpha \sigma )\right]-\ln\dot{\varepsilon }$; (**d**) $\ln\left[\sinh\left(\alpha \sigma \right)\right]\;-{\;T}^{-1}$.

By observing the adjusted flow stress curve, it was found that the spray-formed 7055 forged aluminum alloy reached a stable yield stress state at 0.2% strain, at which point the stress value approaches its peak value. Based on this observation, the steady-state yield stress at a strain ε = 0.4 is selected as the basis for constructing the material constitutive model. From Equation (7), at constant temperature, ln$\dot{\varepsilon }$ exhibits linear relationships with lnσ, σ, and ln[sinh(ασ)]. Therefore, observed Figure 4, the B and β was obtained by calculating the average slope of lnσ-ln$\dot{\varepsilon }$ versus σ-ln$\dot{\varepsilon }$ from the fitted curve. Afterwards, α can be determined by the equation α = β / B. The results indicate that the calculated values are B = 6.14742, β = 0.08841, and α = 0.01438.

By rearranging the formulas applicable to all stress states, the following expressions can be derived:

(8) $$Q={R\left\{\frac{\partial ln\dot{\varepsilon }}{\partial ln\left[\sinh\left(\alpha \sigma \right)\right]}\right\}}_{T}{\left\{\frac{\partial ln\left[\sinh\left(\alpha \sigma \right)\right]}{\partial \left(\frac{1}{T}\right)}\right\}}_{\dot{\varepsilon }}$$

Substitute the stress, temperature, and strain rate values corresponding to a strain of 0.4 into Equation (8), the deformation activation energy Q is obtained through linear regression of $\ln\left[\sinh\left(\alpha \sigma \right)\right]\;-\;{1000\;\times \;T}^{-1}$, yielding Q = 117.853 kJ/mol. The activation energy Q represents a critical parameter characterizing the resistance to plastic deformation in materials [25]. The Q value of pure aluminum (142 kJ/mol) is higher than the Q value obtained in this study [26]. This phenomenon results from the distinctive microstructure generated through the spray forming technique, which consists of a uniform fine-grained structure accompanied by a high dislocation density and finely dispersed second-phase particles [27]. Such a microstructure significantly enhances short-circuit diffusion pathways, including grain boundaries and dislocations. This prioritizes the activation of low-energy barrier deformation mechanisms such as diffusion-assisted dislocation climb and grain boundary slip [28,29,30]. These mechanisms effectively accommodate plastic strain and alleviate localized stress concentration, resulting in the predominance of low-activation-energy processes in macroscopic deformation behavior [31]. Therefore, the overall apparent thermal activation energy is markedly reduced.

According to the work of Zener and Hollomon et al. [32], the Zener–Hollomon parameters defined in Equation (9) be employed to establish a constitutive model relating deformation temperature and strain rate during the hot deformation of alloys.

(9) $$Z=\dot{\varepsilon }\;exp\left(\frac{Q}{RT}\right)$$

By combining Equations (5) and (9), the flow stress constitutive relationship incorporating the Z parameter can be derived, as shown in Equations (10) and (11):

(10) $$Z=\dot{\varepsilon }\;exp\left(Q/RT\right)=A{\left(\sinh\left(\alpha \sigma \right)\right)}^{n}\to lnZ=lnA+n\left(ln\sinh\left(\alpha \sigma \right)\right)$$

(11) $$\sigma =\frac{1}{\alpha }ln\left({\left(\frac{Z}{A}\right)}^{\frac{1}{n}}+{\left({\left(\frac{Z}{A}\right)}^{\frac{2}{n}}+1\right)}^{\frac{1}{2}}\right)$$

**Figure 5:**
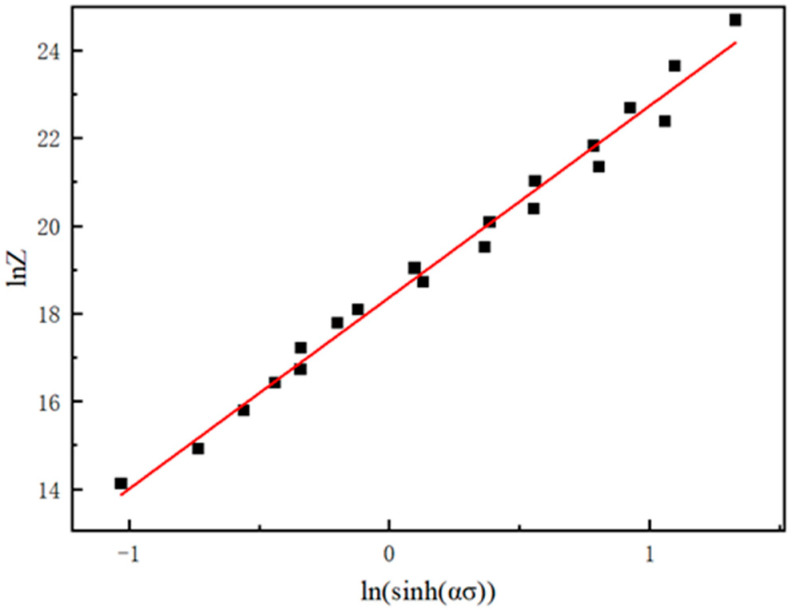
Linear relationship fitting between lnZ and $ln\left[sinh(\alpha \sigma )\right]$.

After fitting the relationship between lnZ and ln[sinh(ασ)] according to Equation (10), the value of A is determined to be 1.847 × 10^8^. The fitting relationship is shown in Figure 5.

Therefore, the Arrhenius constitutive model for spray-formed 7055 as-forged aluminum alloy is given by Equation (12):

(12) $$\sigma =69.53ln\left({\left(\frac{Z}{1.847\times 10^{8}}\right)}^{0.218}+{\left({\left(\frac{Z}{1.847\times 10^{8}}\right)}^{0.436}+1\right)}^{\frac{1}{2}}\right)$$

The established constitutive model above described above fail to show the influence of strain on flow stress, while material constants exhibit significant variations under different strain conditions [31]. Therefore, it is essential to incorporate strain compensation for material parameters during constitutive model formulation. Corresponding material constants were determined at 0.05 true strain intervals following the aforementioned computational procedure. These constants were subsequently fitted using polynomial functions as expressed in Equation (13), where coefficients a, b, c, and d represent the fitted values for material constants α, n, Q, and lnA, respectively.

(13) $$\left\{\begin{array}{c} \;Q=a_{0}+a_{1}\varepsilon +a_{2}\varepsilon^{2}+a_{3}\varepsilon^{3}+a_{4}\varepsilon^{4}+a_{5}\varepsilon^{5}+a_{6}\varepsilon^{6}+a_{7}\varepsilon^{7}+a_{8}\varepsilon^{8}+a_{9}\varepsilon^{9}\\ \alpha =b_{0}+b_{1}\varepsilon +b_{2}\varepsilon^{2}+b_{3}\varepsilon^{3}+b_{4}\varepsilon^{4}+b_{5}\varepsilon^{5}+b_{6}\varepsilon^{6}+b_{7}\varepsilon^{7}+b_{8}\varepsilon^{8}+b_{9}\varepsilon^{9}\\ lnA=c_{0}+c_{1}\varepsilon +c_{2}\varepsilon^{2}+c_{3}\varepsilon^{3}+c_{4}\varepsilon^{4}+c_{5}\varepsilon^{5}+c_{6}\varepsilon^{6}+c_{7}\varepsilon^{7}+c_{8}\varepsilon^{8}+c_{9}\varepsilon^{9}\\ \;n=d_{0}+d_{1}\varepsilon +d_{2}\varepsilon^{2}+d_{3}\varepsilon^{3}+d_{4}\varepsilon^{4}+d_{5}\varepsilon^{5}+d_{6}\varepsilon^{6}+d_{7}\varepsilon^{7}+d_{8}\varepsilon^{8}+d_{9}\varepsilon^{9} \end{array}\right.$$

**Figure 6:**
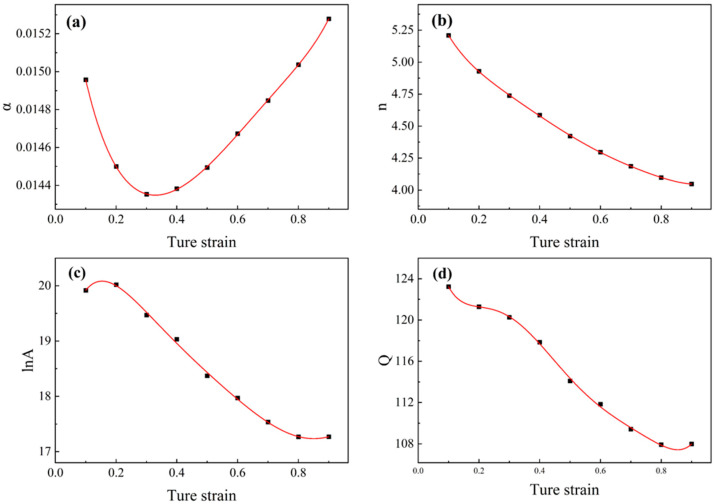
The 9th-order polynomial fitting curve for the relationship between material constants and strain in the Arrhenius constitutive model: (**a**) *α*; (**b**) *n*; (**c**)lnA; (**d**) *Q*.

Figure 6 shows the 9th-order polynomial fitting curve for the material parameters.

Substituting the strain-fitting material parameters obtained above into Equation (11) yields the strain-compensated Arrhenius constitutive model, whose expression is shown in Equation (14).

(14) $$\sigma =\frac{1}{\alpha \left(\varepsilon \right)}ln\left\{{\left(\frac{\dot{\varepsilon }exp\left(\frac{Q\left(\varepsilon \right)}{RT}\right)}{A\left(\varepsilon \right)}\right)}^{1/n\left(\varepsilon \right)}+{\left[{\left(\frac{\dot{\varepsilon }exp\left(\frac{Q\left(\varepsilon \right)}{RT}\right)}{A\left(\varepsilon \right)}\right)}^{2/n\left(\varepsilon \right)}+1\right]}^{\frac{1}{2}}\right\}$$

The correlation coefficient (R) and average absolute relative error (AARE) were employed to quantify the precision of the developed strain-compensated Arrhenius constitutive model. These metrics quantify the differences between predicted flow stresses and their corresponding corrected experimental values, according to Equations (15) and (16).

(15) $$R=\frac{\sum_{i=1}^{N}\left(D_{i}-\bar{D}\right)\left(Y_{i}-\bar{Y}\right)}{\sqrt{\sum_{i=1}^{N}{\left(D_{i}-\bar{D}\right)}^{2}\sum_{i=1}^{N}{\;\left(Y_{i}-\bar{Y}\right)}^{2}}}$$

(16) $$AARE=\frac{1}{N}\sum_{i=1}^{N}\left|\frac{D_{i}-Y_{i}}{D_{i}}\right|$$

Here, N denotes the total number of samples; D_i_ and D represent the experimentally measured stress value and its mean, respectively; and Y_i_ and Y correspond to the predicted stress value and its mean.

**Figure 7:**
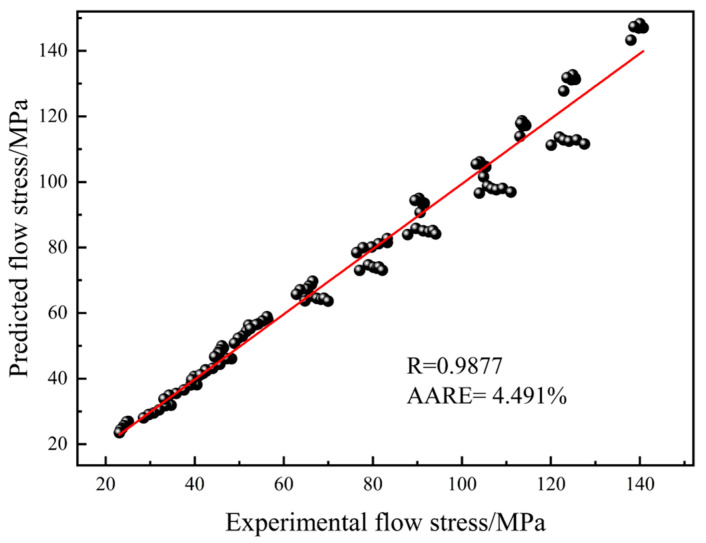
Correlation between predicted values of strain modification constitutive equation and experimental values.

As shown in Figure 7, after integrating the predicted values from Equation (14) with the experimentally calibrated data adjusted for friction and temperature, the R was determined to be 0.9877, and the AARE was found to be 4.491%.

### 3.3. Hot Processing Map

Hot processing maps serve as critical tools for evaluating material workability, enabling prediction of optimal processing windows and identification of potential defect-prone regions. Through the integration of thermal processing maps with microstructural evolution analysis, material processing parameters can be optimized to achieve superior process design [33,34].

Currently established thermal processing methodologies include atomic model-based maps, kinetic models, and the Dynamic Materials Model (DMM) processing maps [35]. This study employs the DMM-based hot processing map, which integrates a power dissipation map with a plastic instability map. Within this framework, the hot working process is treated as a system for energy dissipation. The total input power P during plastic flow comprises the work done by plastic deformation and the work consumed by microstructural changes, as given by Equation (17) [36]:

(17) $$P=\int_{0}^{\bar{\varepsilon }}\bar{\sigma }d\dot{\bar{\varepsilon }}+\int_{0}^{\bar{\sigma }}\dot{\bar{\varepsilon }}d\bar{\sigma }=G+J$$

Here, G represents the energy consumed by plastic deformation, and J corresponds to the energy dissipation resulting from microstructural changes.

Under constant deformation conditions, the variation of rheological stress with strain rate can be described by the strain rate sensitivity index m [37], defined as follows:

(18) $$m={\left[\frac{\partial J}{\partial G}\right]}_{\varepsilon ,T}={\left[\frac{\dot{\varepsilon }\partial J}{\partial G}\right]}_{\varepsilon ,T}={\left[\frac{\partial \left(ln\sigma \right)}{\partial \left(ln\dot{\varepsilon }\right)}\right]}_{\varepsilon ,T}$$

Here, K denotes a material constant governed by deformation temperature and material state.

During the hot deformation of metals, the strain rate sensitivity index (m) demonstrates thermally driven variations, generally falling between 0 and 1 under varying temperatures and strain rates. When the m = 1, G and J become equivalent, indicating that the system is in an ideal linear dissipative state, at which point J reaches its maximum value. This condition can be described by the following mathematical expression:

(19) $$J_{max}=\frac{1}{2}\sigma \dot{\varepsilon }$$

To quantitatively characterize the relationship between the energy dissipated during microstructural evolution and the total energy consumed by deformation in the thermal deformation process of metals, a power dissipation efficiency factor is defined, and its calculation is given by Equation (20) [38]:

(20) $$𝜂=\frac{J}{J_{max}}=\frac{2m}{1+m}$$

**Figure 8:**
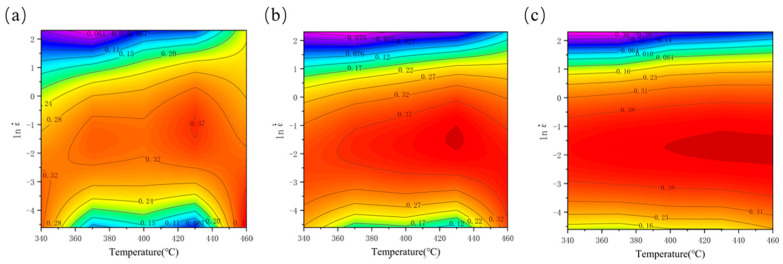
Power dissipation maps of spray-formed 7055 as-forged aluminum alloy under different strains: (**a**) 0.3; (**b**) 0.6; (**c**) 0.9.

By calculating the power dissipation efficiency factor (η), a power dissipation map is constructed to examine the influence of temperature and strain rate on η at a specific strain level. In such maps, the contour values correspond to the power dissipation efficiency, with dark red areas indicating regions of higher efficiency under the corresponding processing parameters. In high-efficiency regions, a greater proportion of energy is consumed by microstructural changes, promoting the dominance of softening mechanisms. Thereby, favorable deformation microstructures are formed, leading to enhanced workability. As a result, favorable deformed microstructures are formed, and workability is improved [32]. As illustrated in Figure 8, the power dissipation maps across various strains reveal that high-efficiency regions (η > 30%) are predominantly located within the strain rate interval of 0.05 s^−1^–1 s^−1^.

However, elevated power dissipation efficiency can also correspond to material flow instability under these processing conditions. Consequently, the corresponding parameter regimes should be avoided.

To quantitatively assess the flow stability during hot deformation, Prasad et al. [39] introduced an instability criterion ξ, defined as:

(21) $$\xi =\frac{\partial ln\frac{m}{1+m}}{\partial ln\dot{\varepsilon }}+m<0$$

Within the coordinate system of strain rate versus deformation temperature, a contour map of the ξ at a given strain constitutes the processing instability map. When ξ < 0, the material undergoes plastic instability [40].

**Figure 9:**
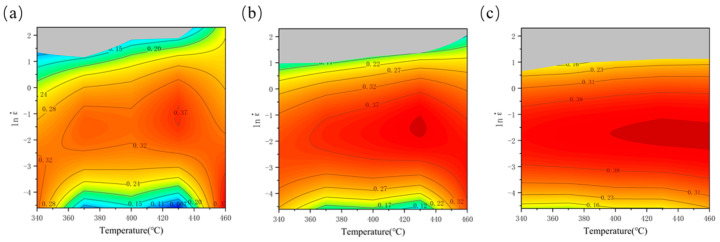
Processing maps of spray-formed 7055 as-forged aluminum alloy under different strains; (**a**) 0.3, (**b**) 0.6, and (**c**) 0.9.

The hot processing map was developed by superimposing the instability map onto the power dissipation map. Figure 9 depicts the hot processing maps of the spray-formed 7055 forged aluminum alloy at strain levels of 0.3, 0.6, and 0.9. Contour values denote power dissipation efficiency. And shaded areas identify unstable regions. Notably, the maps show high consistency across varying strains. All maps display extensive instability regions, the area of which increases with accumulating strain. These instability regions are predominantly located in the upper section of the maps. As illustrated, the peak power dissipation efficiency for this alloy occurs at a strain of 0.9. Under this condition, the area exhibiting higher power dissipation efficiency is mainly observed between 400–460 °C and 0.08–0.37 s^−1^. Furthermore, the contour lines within this region are not densely packed, indicating a relatively stable processing window. Conversely, the instability regions are predominantly concentrated within the range of 340–460 °C/1.7–10 s^−1^. This observed instability can be attributed to the following mechanisms: Under high strain rate conditions, dislocation motion is impeded, and dynamic softening mechanisms, including DRV and DRX, are inhibited due to insufficient time for their activation. Even at elevated deformation temperatures, dislocations moving at high velocities may be unable to bypass obstacles (e.g., via climb) rapidly enough, leading to dislocation pile-ups. Concurrently, the dislocation density accumulates rapidly, resulting in pronounced work hardening. The absence of effective softening mechanisms to counteract this hardening causes a sharp increase in material stress. Consequently, micro-cracks or voids readily nucleate at defects or stress concentration sites and propagate rapidly. This ultimately leads to material instability or localized deformation.

### 3.4. Microstructure Evolution

#### 3.4.1. Microstructural Evolution with Strain Rate

**Figure 10:**
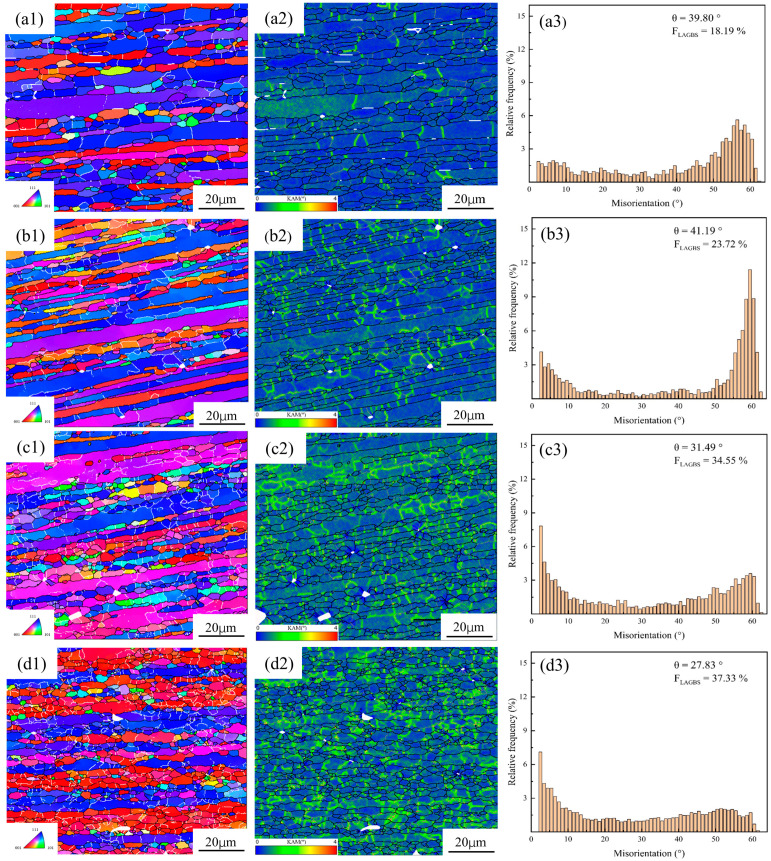
IPF, KAM and MAD maps of the spray-formed 7055 as-forged aluminum alloy deformed at 430 °C under different strain rates: (**a1**,**a2**,**a3**) 0.01 s^−1^; (**b1**,**b2**,**b3**) 0.1 s^−1^; (**c1**,**c2**,**c3**) 1 s^−1^; (**d1**,**d2**,**d3**) 10 s^−1^.

Figure 10 displays IPF, KAM, and MAD maps of the spray-formed 7055 as-forged aluminum alloy, deformed at 430 °C with strain rates ranging from 0.01 s^−1^ to 10 s^−1^. The microstructures observed under different strain rates correspond well with the thermal processing map established earlier.

As illustrated in Figure 10(a1–a3), at 0.01 s^−1^, uniform chains of equiaxed grains form around the original grain boundaries. This morphology is characteristic of discontinuous dynamic recrystallization (DDRX) [41]. The recrystallized grains are relatively coarse and exhibit significant orientation differences compared to the original grains. Concurrently, the proportion of LAGBs is low under this strain rate, and the grain boundaries appear relatively straight. These features collectively indicate a high degree of recrystallization completeness. Sufficient DRV is achieved at low strain rates. This enables grain boundary migration driven by boundary energy, consistent with the DDRX mechanism. New grains nucleate primarily through grain boundary bulging and subsequently grow, developing into HAGBs. Furthermore, the mean KAM value is relatively small under this condition, with only a limited number of dislocations distributed within the grain interiors. This observation arises because dislocations generated by deformation have sufficient time for rearrangement and annihilation processes. Consequently, plastically deformed regions become uniformly distributed throughout the matrix.

As illustrated in Figure 10(b1–b3), at 0.1 s^−1^, compared to the lower rate of 0.01 s^−1^, a moderate increase is observed in both the proportion of LAGBs and the KAM values. Concurrently, grain refinement occurs, accompanied by the formation of a limited number of subgrain structures and the development of a weak texture. Despite these changes, HAGBs remain predominant. Furthermore, the crystallographic orientations of the grains exhibit a predominantly random distribution. These microstructural features suggest that DDRX persists as the dominant mechanism. This phenomenon primarily stems from the increased strain rate. The higher rate elevates the recrystallization nucleation rate while simultaneously promoting greater dislocation accumulation. The refinement of recrystallized grains results from this factor combination, as will be discussed subsequently.

As illustrated in Figure 10(c1–c3), at 1 s^−1^, a significant decrease in the average misorientation angle (θ) is observed. Concurrently, both the proportion of LAGBs and the average KAM value exhibit a pronounced increase. These changes collectively indicate a substantial rise in the presence of subgrain structures within the microstructure. The microstructure demonstrates pronounced heterogeneity, characterized by a mixed distribution of deformed grains retaining their original orientations and recrystallized grains. Furthermore, the dislocation distribution becomes markedly inhomogeneous. Deformed grains exhibit elevated KAM values within their interiors, signifying high local lattice distortion and dislocation density. In contrast, recrystallized grains display low internal KAM values. Regions of high KAM correspond directly to areas containing subgrain structures. This microstructural evolution is attributed to the high strain rate condition. Under such conditions, the dislocation multiplication rate exceeds the rate of DRV. Consequently, dislocation density accumulates rapidly. This triggers the activation of CDRX, whereby dislocations rearrange to form subgrains. These subgrains progressively evolve into HAGBs through the absorption of dislocations and boundary migration. However, at this strain rate, the CDRX process remains incomplete. This incomplete transformation results in a significantly higher proportion of LAGBs compared to lower strain rates. The observed decrease in the characteristic misorientation angle (θ) further corroborates the extensive presence of subgrain boundaries (i.e., LAGBs) within the microstructure.

As illustrated in Figure 10(d1–d3), at 10 s^−1^, a further decrease in the average misorientation angle (θ) and a continued rise in the proportion of LAGBs are observed. These trends signify a heightened prevalence of subgrain boundaries and a concomitant reduction in the overall degree of recrystallization. The microstructure is predominantly characterized by a deformed matrix, featuring distinctly elongated grains. Localized regions contain fine recrystallized grains. Furthermore, the crystallographic texture exhibits pronounced intensity, dominated by strong <111> and <001> fiber components. As shown in Figure 10(d2), the KAM values are generally elevated across the microstructure, particularly within the interiors of deformed grains. Notably, localized zones of exceptionally high KAM are present, indicative of regions possessing significantly elevated stored energy. These high-energy regions are more conducive to the generation of fine grains and may facilitate localized recrystallization [13,42]. The ultra-high strain rate is the main cause of this behavior. The increased rate substantially raises the deformation activation energy, leading to intensified dislocation multiplication and accumulation. Consequently, CDRX becomes the dominant mechanism. However, the persistently high LAGB fraction and the low average θ value unambiguously indicate that the material remains in a state of incomplete recrystallization, retaining a substantial density of subgrain structures [43]. Additionally, adiabatic heating induced by the rapid plastic deformation may promote DRV and localized recrystallization events. Nevertheless, the overall extent of recrystallization remains low under these extreme deformation conditions.

#### 3.4.2. Microstructural Evolution with Deformation Temperature

**Figure 11:**
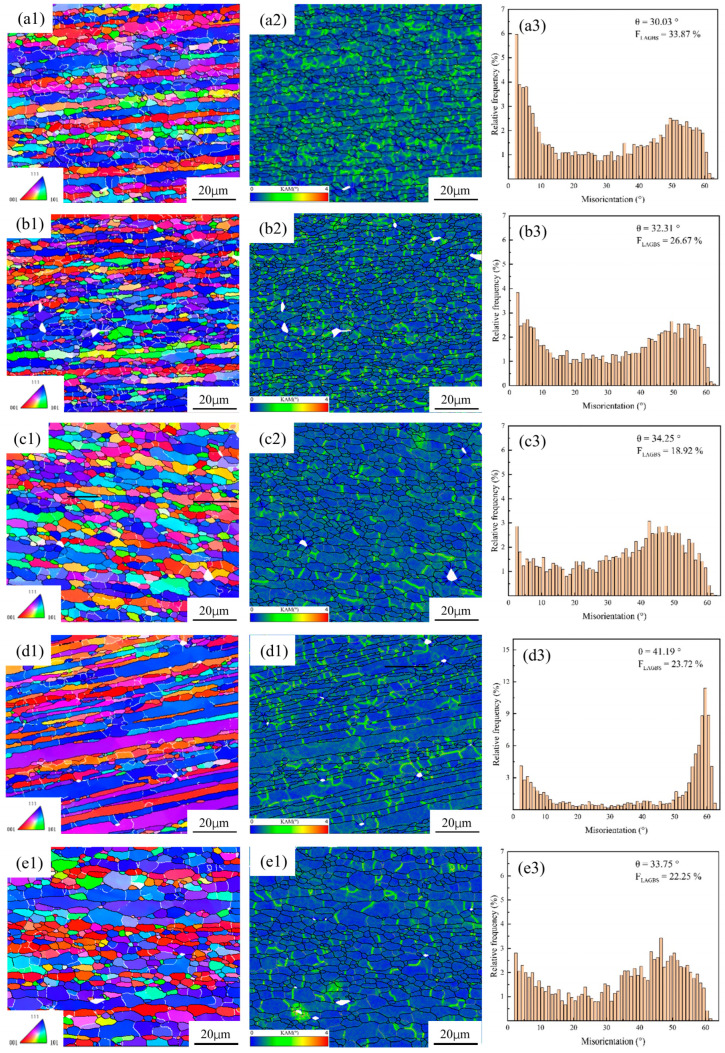
IPF, KAM and MAD maps of spray-formed 7055 as-forged aluminum alloy deformed at a strain rate of 0.1 s^−1^ under different temperatures: (**a1**,**a2**,**a3**) 340 °C; (**b1**,**b2**,**b3**) 370 °C; (**c1**,**c2**,**c3**) 400 °C; (**d1**,**d2**,**d3**) 430 °C; (**e1**,**e2**,**e3**) 460 °C.

Figure 11 presents the IPF, KAM and MAD maps of spray-formed 7055 forged aluminum alloy after deformation at multiple temperatures (340 °C, 370 °C, 400 °C, 430 °C, and 460 °C) and a strain rate of 0.1 s^−1^. Analysis of the figures reveals that within the temperature range of 340 °C to 460 °C, the average misorientation angle rises, whereas the fraction of LAGBs declines. Concurrently, chain-like arrays of equiaxed grains progressively emerge along the boundaries of the original grains. These microstructural evolution characteristics indicate the occurrence of DRX.

According to Figure 11(a1–a3), at 340 °C, partial grain refinement is observed, with a high proportion of LAGBs reaching 33.87%. However, a significant number of deformed grains persist, resulting in a non-uniform microstructure distribution. This inhomogeneity arises because dislocations reorganize primarily through slip and climb [44,45], forming subgrain structures. The relatively low deformation temperature is insufficient to fully promote complete recrystallization. Consequently, DRV remains the predominant mechanism, leading to a hybrid microstructure comprising partially formed subgrain and retained deformed grains.

According to Figure 11(b1–b3), at a deformation temperature of 370 °C, the average misorientation angle (θ) increases to 32.31°, with the proportion of LAGBs decreasing and the orientation gradient diminishing. This indicates that partial subgrain are transforming into HAGBs through mechanisms such as coalescence or rotation, signifying the initiation of CDRX. Consequently, the proportion of recrystallized grains rises; however, the deformed microstructure remains predominant. With the deformation temperature increasing to 400 °C, the original deformed grains are significantly reduced (Figure 11(c1–c3)).The misorientation angle distribution exhibits a bimodal character. Concurrently, grain boundaries become straighter, grain orientations show enhanced randomness, and the texture weakens. These microstructural changes suggest a transition from CDRX towards DDRX. Elevated temperatures enhance grain boundary migration, thereby accelerating recrystallization and thus promoting the development of more equiaxed grains.

According to Figure 11(d1–d3), under the condition of 430 °C, the coexistence of CDRX and DDRX is evidenced by the emergence of both non-continuous and continuous HAGBs within grain interiors. The observed increase in the proportion of local LAGBs is attributed to the phenomenon of subgrain coalescence. However, the rate of subgrain coalescence lags behind dislocation annihilation, resulting in the incomplete transformation of some LAGBs into HAGBs. Concurrently, elevated temperature deformation accelerates dislocation migration, facilitating the rearrangement of dislocations via slip and climb mechanisms, which promotes the formation of new LAGBs. At grain boundary triple junctions, a significant population of fine-grained equiaxed crystals persists. This is due to the concentration of strain at these triple junctions, where high dislocation densities promote dynamic recrystallization nucleation, resulting in the formation of these fine grains. Furthermore, solute atoms (Mg, Cu) begin to detach from grain boundaries, reducing the impedance caused by second-phase particles. This induces a sharp increase in grain boundary migration rate [41]. Nevertheless, grain boundary migration occurs non-uniformly; localized rapid migration results in certain regions being left behind during the recrystallization process.

According to Figure 11(d1–d3), under the condition of 460 °C, a bimodal grain size distribution emerges, featuring a mixture of both coarse and fine grains. Concurrently, grain boundaries exhibit curvature and localized serration. At this temperature, solute atoms (Mg, Cu) migrate back towards the grain boundaries, inducing a distinct solute drag effect [46]. This enhanced drag considerably impedes grain boundary movement. The associated decrease in the boundary migration angle (θ) thereby leads to the visible serration of the grain boundaries. Furthermore, this regime is accompanied by abnormal grain growth, driven by the inherent reduction in grain boundary energy associated with the migration process.

According to Figure 11(a2), at 340 °C, KAM maps reveal relatively high KAM values, indicative of significant local plastic deformation within the microstructure. Concurrently, LAGBs are uniformly distributed throughout the grain interiors. This microstructural state predominantly corresponds to the recovery stage, where dislocations undergo rearrangement without achieving complete recrystallization. With increasing deformation temperature, KAM values decrease, indicating reduced dislocation density and the onset of recrystallization accompanied by new grain formation. This occurs as elevated thermal energy facilitates dislocation glide and climb, thereby inducing recrystallization. However, at 430 °C (Figure 11(d2)), an anomalous increase in the average KAM value is detected. This observation further corroborates the earlier proposition that solute atoms detach from grain boundaries, diminishing the pinning effect exerted by second-phase particles. The resultant reduction in pinning resistance leads to a non-uniform acceleration in grain boundary migration rate. Consequently, dislocation annihilation becomes heterogeneous, leaving localized regions exhibiting elevated dislocation density, as reflected in the higher average KAM

## 4. Conclusions

This work employed isothermal hot-press tests to acquire experimental true stress–strain data for spray-formed 7055 as-forged aluminum alloy over a range of processing parameters. Based on this dataset, established an Arrhenius-type constitutive model. Furthermore, through comprehensive microstructural characterization, the research provides in-depth insights into the mechanisms by which deformation temperature and strain rate govern microstructural evolution. The principal findings are summarized as follows:

(1) An Arrhenius constitutive model was established based on flow stress data corrected for friction and temperature. The correlation coefficient (R) between the rheological stress predictions from this model and experimental values is 0.9877, with an average absolute relative error (AARE) of 4.491%.

(2) Established the processing map by integrating the three-dimensional instability criterion with the power consumption efficiency distribution. The optimal processing window was determined to be 400–460 °C/0.08–0.37 s^−1^. Instability regimes are predominantly concentrated within the range of 340–460 °C/1.7–10 s^−1^.

(3) The deformation temperature affects the recrystallization mechanism by regulating the state of solute atom polarization: dynamic reversion (DRV) and continuous dynamic recrystallization (CDRX) dominate at 360–400 °C; discontinuous dynamic recrystallization (DDRX) dominates at 430 °C; and at 460 °C, solute regression and grain-boundary energies jointly promote the growth of anomalous grains and the formation of a mixed-crystalline organization.

(4) The strain rate significantly affects the dynamic softening mechanism: DDRX predominates in the low strain rate regime (≤0.01 s^−1^); CDRX predominates in the high strain rates (≥1 s^−1^); at 10 s^−1^, adiabatic heating causes a heightened gradient of dislocation density within the shear zone, intensifying local deformation and promoting microstructural instability.

## Figures and Tables

**Table 1:** Measured composition for spray-formed 7055 as-forged aluminum alloy (wt. %).

Zn	Mg	Cu	Zr	Si	Fe	Al
8.25	2.03	2.25	0.12	0.05	0.04	other

## Data Availability

The original contributions presented in this study are included in the article. Further inquiries can be directed to the corresponding author.
